# Position of the Hyoid Bone and Dimension of Nasopharynx and Oropharynx after Occlusal Splint Therapy and Physiotherapy in Patients Diagnosed with Temporomandibular Disorders

**DOI:** 10.3390/jcm11174939

**Published:** 2022-08-23

**Authors:** Marcin Derwich, Elzbieta Pawlowska

**Affiliations:** 1 ORTODENT, Specialist Orthodontic Private Practice in Grudziadz, 86-300 Grudziadz, Poland; 2 Department of Orthodontics, Medical University of Lodz, 90-419 Lodz, Poland

**Keywords:** hyoid bone, oropharynx, nasopharynx, occlusal splint therapy, physiotherapy, temporomandibular joints, temporomandibular disorders, obstructive sleep apnea

## Abstract

*Background*: The aim of the study was to assess the position of the hyoid bone, as well as the width of the nasopharynx and oropharynx after occlusal splint therapy combined with physiotherapy in patients diagnosed with temporomandibular disorders (TMD). *Methods*: This was a clinical trial study. The study group consisted of 40 patients diagnosed with TMD, who were qualified for the treatment combining physiotherapy and occlusal splint therapy. Hyoid bone position as well as the width of the nasopharynx and oropharynx were assessed in lateral cephalograms taken before and after the end of the treatment. There were 15 generally healthy participants included into the control group, who had taken lateral cephalograms twice within the period of 1 to 2 years and did not receive any occlusal treatment in the meantime. *Results*: The position of the hyoid bone was significantly lowered and the dimension of the lower part of the oropharynx was significantly decreased after the end of the long-term occlusal splint therapy combined with physiotherapy in patients diagnosed with TMD. *Conclusions*: Long-term occlusal splint therapy combined with physiotherapy affected the position of the hyoid bone and the dimension of the lower part of the oropharynx.

## 1. Introduction

The hyoid bone is the only bone within the human body that is not directly articulated to any other bone. It is localized within the neck, below the mandible, and above the larynx at the level of C3 (third cervical vertebrae). The hyoid bone consists of the body, the greater horns, and the lesser horns [1,2]. There are several muscles and ligaments attached to the hyoid bone, which link the hyoid bone with other anatomical structures, including: mandible (digastric muscle, mylohyoid muscle, geniohyoid muscle), temporal bone (digastric muscle, stylohyoid muscle, stylohyoid ligament), tongue (hyoglossus, tongue intrinsic muscles), sternum (sternohyoid muscle), scapula (omohyoid muscle), larynx (thyrohyoid muscle, thyrohyoid ligament, hyoepiglottic ligament), and cervical spine (middle pharyngeal constrictor muscle) [1].

Due to the fact that the hyoid bone is attached to the above-listed anatomic structures via several different types of muscles or ligaments, it is involved in swallowing, breathing, mouth opening, and speech [1,2,3,4,5].

The vertical position of the hyoid bone may be changed depending on the curve of cervical spine, as well as the tension of the supra- and infrahyoid muscles. The digastric muscle, mylohyoid muscle, and geniohyoid muscle are attached to the mandible and therefore make the position of the mandible and the hyoid bone interrelated.

Temporomandibular disorders (TMD) are part of a broad term which encompasses different types of musculoskeletal diseases, localized in the area of the temporomandibular joints (TMJs), masticatory muscles, and adjacent tissues [6]. The etiology of TMD is complex and therefore patients suffering from temporomandibular disorders (TMD) most commonly require an interdisciplinary approach. The conservative management of TMD includes: counseling, occlusal splint therapy, physiotherapy, and pharmacotherapy. Occlusal splint therapy and physiotherapy are most often considered to be the first-line therapy in patients diagnosed with TMD [6,7]. It has been found that long-term occlusal splint therapy combined with physiotherapy leads to the posterior rotation of the mandible, as well as to the posterior rotation of the cranium; decreases in the width of the C1-C2 functional space; and finally leads to the restoration of the cervical lordosis [8].

It seems reasonable that changes in the position of the cranium, as well as the mandible, may affect the position of the hyoid bone. The position of the hyoid bone is linked to the breathing disturbances. It has been found that a lowered position of the hyoid bone may be related to obstructive sleep apnea (OSA) [9,10]. Therefore, changes in the position of the hyoid bone may be either beneficial or harmful for the OSA patients, depending on whether the hyoid bone moves up or down. However, so far, nothing is known about if the long-term occlusal splint therapy affects the position of the hyoid bone, as well as the width of the nasopharynx and oropharynx.

The aim of the study was to assess the position of the hyoid bone, as well as the width of the nasopharynx and oropharynx after occlusal splint therapy combined with physiotherapy in patients diagnosed with temporomandibular disorders (TMD).

The null hypothesis was that the long-term occlusal splint therapy and physiotherapy do not affect the position of the hyoid bone, as well as the width of the nasopharynx and oropharynx.

## 2. Materials and Methods

This was a clinical trial study. The study was approved by the Medical Board Ethical Committee of Regional Medical Chamber in Gdansk, Poland (KB-17/21) and was conducted with the ethical principles of the World Medical Association Declaration of Helsinki. All patients received and signed an informed consent form.

This manuscript is a further evaluation of the changes that occur in patients diagnosed with TMD after long-term occlusal splint therapy and physiotherapy. So far, we have described the position of the mandibular condyle within the glenoid fossa [11], and the craniovertebral and the craniomandibular changes after long-term occlusal splint therapy and physiotherapy [8].

### 2.1. Participants

All of the participants were the patients belonging to the specialist orthodontic private practice in Grudziadz (Poland).

The study group consisted of patients who had been diagnosed with TMD on the basis of the Diagnostic Criteria for Temporomandibular Disorders (DC/TMD) [12]. The inclusion criteria were: the diagnosis of TMD, age between 18 and 65 years old, and willingness to take part in the study. There were also a few exclusion criteria: patients who underwent oncological treatment; previous traumas in the area of head and neck; previous TMJ surgery, orthognathic surgery, and neck surgery; rheumatological diseases; pregnancy; and history of previous orthodontic treatment.

The control group consisted of healthy patients without symptoms of TMD. Patients from the control group underwent the process of orthodontic diagnosis twice because they decided to start orthodontic treatment more than 1 year after the initial diagnosis had been performed. The period of time between the first and second orthodontic examination lasted from 1 to 2 years.

### 2.2. Investigation

All of the participants underwent an initial extraoral and intraoral examination. The extraoral examination included palpation of the temporomandibular joints (TMJs), and palpation of the head and neck muscles. After the first examination had been performed, the lateral cephalograms were taken in the natural head position, and in maximum intercuspation.

Patients diagnosed with TMD were referred to the physiotherapist for the initial physiotherapy of the area of the head and neck (once a week for 5 consecutive weeks). The full protocol of physiotherapy was described in detail in our former studies [8,11]. All of the TMD patients received also some exercises (Rocabado’s 6 × 6 exercises) [13] to be performed as autotherapy.

After completion of the initial series of physiotherapy, the occlusal splints were manufactured for all of the TMD patients. The acrylic occlusal splints were: one-piece, hard, flat, with canine and anterior guidance, and were covering the full upper arch. All of the teeth from the lower arch were supposed to have one contact point with the occlusal surface of the occlusal splint. The occlusal splint therapy lasted 6 months and it was supported by regular physiotherapy. The full protocol of check-ups and adjustments of occlusal splints has been described in our previous studies [8,11]. Having completed the 6-month period of simultaneous occlusal splint therapy and physiotherapy, all of the TMD patients underwent a second extraoral and intraoral examination. During the second examination, lateral cephalograms were taken again. Those X-rays were taken without occlusal splints. All of the examinations were performed by one of the researchers (MD).

### 2.3. Outcome Measures

The primary outcome was to assess the changes of the hyoid bone position in relation to the cranium, maxilla, and mandible after occlusal splint therapy combined with physiotherapy in patients diagnosed with TMD.

The secondary outcome was to assess the changes in the width of the nasopharynx and oropharynx after occlusal splint therapy combined with physiotherapy in patients diagnosed with TMD.

Table 1 presents the reference points, lines, and angles used in the cephalometric analysis to assess the position of the hyoid bone and the width of the nasopharynx and oropharynx after occlusal splint therapy combined with physiotherapy in patients diagnosed with TMD [14,15].

**Figure 1:**
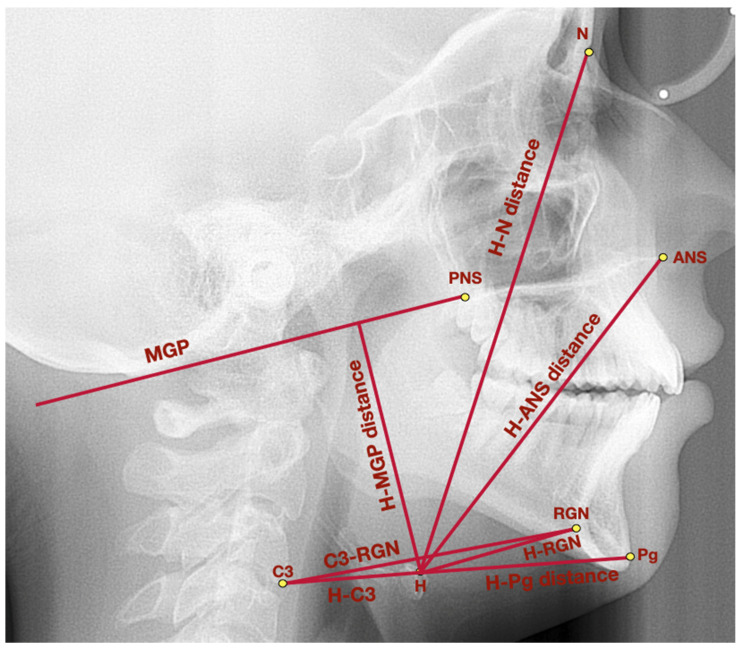
Lateral cephalogram with marked points, lines, and angles presented in Table 1 used to assess the hyoid triangle and the hyoid bone topography. ANS—anterior nasal spine, C3—third cervical vertebrae, H—hyoidale, MGP—McGregor’s Plane, N—nasion, Pg–pogonion, PNS—posterior nasal spine, RGN—retrognathion.

Figure 1 presents a lateral cephalogram with the marked points, lines, and angles presented in Table 1 used to assess the hyoid triangle and the hyoid bone topography.

**Figure 2:**
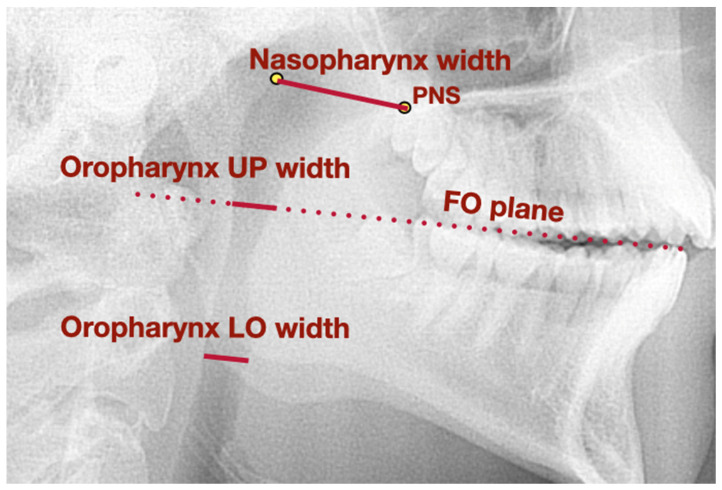
Lateral cephalogram with marked points, lines, and angles presented in Table 1 used to assess airways dimension. FO plane—functional occlusal plane, LO—lower, PNS—posterior nasal spine, UP—upper.

Figure 2 presents a lateral cephalogram with the marked points, lines, and angles presented in Table 1 used to assess the airways dimension.

### 2.4. Statistical Analysis

To perform all data analyses, Statistica 13.0 software (Dell Inc., Aliso Viejo, CA, USA) was used. There were several values calculated: mean differences, standard deviations, 95% confidence interval (95% CI), and mean percentage changes between the values obtained before and after the end of the treatment. To check whether the differences before and after the end of the treatment were statistically significant, the following tests were applied: the T-Student test, U Mann–Whitney test, and Wilcoxon test. The statistical significance level was set at *p* = 0.05.

## 3. Results

### 3.1. Flow of Participants

There were 44 patients diagnosed with TMD who were qualified to take part in the study. Four patients did not complete the whole period of occlusal splint therapy and therefore they were excluded from the study. Finally, there were 40 patients who completed the prescribed treatment. The average age of TMD patients was: 26.1 ± 11.6 years old (range: 18–61 years old). There were 15 generally healthy patients included into the control group. The average age of patients from the control group was: 31.3 ± 12.9 years old (range: 18–58 years old). The distribution of sexes was the same in both the control group and the study group, namely: 80.0% of participants were women and 20.0% were men.

**Figure 3:**
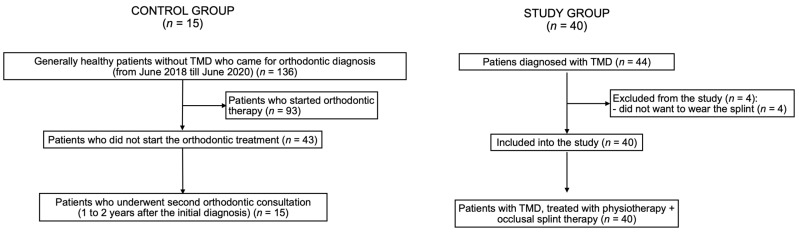
Flow of the participants during the study. TMD—temporomandibular disorders.

Figure 3 presents the flow of the participants during the study.

The diagnoses on the basis of the DC/TMD among the examined patients from the study group were presented in our former studies [8,11]. The most common diagnosis on the basis of DC/TMD was myalgia, which was diagnosed in 29 patients from the study group (72.5%).

### 3.2. Research Question

There were some significant differences in the examined parameters regarding the position of the hyoid bone and the width of the nasopharynx during the initial examination between the study group and control group. The distance between C3 and RGN was significantly larger in the control group (70.6 ± 8.3 mm) compared to the study group (64.1 ± 8.6 mm) (*p* = 0.0104). The distance between hyoidale and pogonion points was significantly larger in the control group (51.9 ± 6.6 mm) compared to the study group (47.8 ± 6.1 mm) (*p* = 0.0256). Finally, the nasopharynx width was significantly larger in the control group (23.2 ± 3.2 mm) compared to the study group (20.3 ± 3.4 mm) (*p* = 0.0241).

Table 2 presents the comparison of the examined parameters regarding the hyoid bone position and the width of the nasopharynx and oropharynx during the initial examination between the study group and control group.

Within the control group, there were no statistically significant differences between the first and the second examination regarding: the hyoid triangle, topography of the hyoid bone, and the airways dimension.

Table 3 presents the assessment of the changes in the hyoid bone position and the width of the nasopharynx and oropharynx that occurred between the first and the second orthodontic examination in the control group.

Within the study group, the average distances between H and C3, H and RGN, as well as C3 and RGN were similar before and after the end of the treatment. However, the average height of the hyoid triangle (H-H’ distance) significantly increased by 41.82% from 5.5 ± 5.4 mm (95% CI 4.2 to 7.1) to 7.8 ± 6.4 mm (95% CI 5.8 to 9.9) (*p* < 0.0001) after the treatment. The average distance between H and MGP increased by 5.86%, the average distance between H and N increased by 3.38%, and the average distance between H and the anterior nasal spine increased by 4.6% after the end of the treatment. Contrary to those changes, the average distance between H and Pg did not change significantly. Finally, the average width of the lower part of the oropharynx significantly decreased by 10.75% from 9.3 ± 2.9 mm (95% CI 8.4 to 10.0) to 8.3 ± 2.6 mm (95% CI 7.6 to 9.0) (*p* = 0.0104). The average width of the upper part of the oropharynx decreased by 3.0%, and the average width of the nasopharynx decreased by 0.5% after the end of the therapy.

Table 4 presents changes that occurred in the hyoid bone position and the width of the nasopharynx and oropharynx after occlusal splint therapy combined with physiotherapy in patients diagnosed with TMD.

## 4. Discussion

This is the first study which prospectively analyzed the position of the hyoid bone, as well as the width of the nasopharynx and oropharynx in patients with TMD after the conservative treatment, including occlusal splint therapy combined with physiotherapy.

Having compared the initial measurements between the examined groups, we have observed that patients diagnosed with TMD presented a significantly lower distance between C3 and retrognathion, a significantly lower distance between hyoidale and pogonion, and finally a significantly lower nasopharynx width comparing to the generally healthy participants.

The relationship between TMD and hyoid bone position has been investigated by several authors. Zhou et al. [16] analyzed the position of the hyoid bone in three groups: normal group (patients with normal morphology of the mandibular condyles), indeterminate for osteoarthrosis (condyles diagnosed with articular surface flattening or subcortical sclerosis, and at the time without any other symptoms), and finally, patients with osteoarthrosis (OA). The authors noticed that the hyoid bone was positioned closer to the cranium and mandible in adult patients with TMJ OA. Moreover, they also found that the distance between C3 and retrognathion was significantly reduced in all patients diagnosed with TMJ OA compared to healthy participants. These observations stay in agreement with our results. Ekici et al. [17] found that the distances between hyoidale and Basion, as well as between hyoidale and the sella-nasion line were significantly reduced in patients with TMD comparing to the control group. Contrary to the previously mentioned studies, Andrade et al. [18] did not find any statistically significant differences regarding the horizontal as well as vertical position of the hyoid bone between patients diagnosed with TMD and the control group. Câmara-Souza et al. [19] compared the height of the hyoid triangle (H-H’ distance) in patients with and without TMD. The authors did not find any differences between the examined groups. This observation is consistent with our results, as we also did not observe any significant differences between the groups during the initial examination. Câmara-Souza et al. [19] concluded that there was no relationship between TMD and craniocervical posture, including the position of the hyoid bone. To sum up, there is not enough scientific evidence to confirm the relationship between the position of the hyoid bone and the occurrence of TMD.

According to our study, six-month occlusal splint therapy combined with physiotherapy led to the lowering of the hyoid bone position. Having analyzed the lateral cephalograms, we have noticed that the average values of the height of the hyoid triangle (H-H’ distance) significantly increased in patients with TMD after the end of the treatment. Moreover, the average distances between the hyoidale point and different points or planes localized within the cranium (McGregor’s Plane, nasion, anterior nasal spine) also significantly increased after the end of the treatment. The average distance between hyoidale and pogonion did not change significantly after the end of the treatment. This is because the mandible rotated downward and backward during the long-term occlusal splint therapy, which we have described in detail in our former study [8]. When the pogonion moved down and back, and the hyoid bone at the same time moved down, the distance between those two anatomic structures remained nearly the same.

Interestingly, we have also observed after the end of the treatment a significant decrease in the average width of the lower part of the oropharynx, measured at the level of the mandibular angle. The average width of the nasopharynx and upper part of the oropharynx (measured at the level of the functional occlusal plane) did not change significantly after the end of the treatment. This means that the long-term occlusal splint therapy combined with physiotherapy led to the simultaneous lowering of the hyoid bone position, decreasing the width of the lower part of the oropharynx, and to the rotation of the mandible downward and backward [8].

Changes in the position of the hyoid bone and the width of the oropharynx (basically its lower part) did not occur within the control group. This means that both the position of the hyoid bone and the width of the oropharynx did not change with time within the 2-year period of observation.

According to the available literature, an inferior position of the hyoid bone is highly correlated with obstructive sleep apnea (OSA) [9,10]. It has been noticed that the lowered position of the hyoid bone affects the dimension of the airways by increasing the risk of the so-called pharyngeal collapse [10]. Young et al. [20] found that the severity of obstructive sleep apnea hypopnea syndrome (OSAHS) is correlated with the vertical position of the hyoid bone. The authors assessed the vertical position of the hyoid bone by the distance between the hyoid bone and sella turcica point and noticed that the distance of 120 mm distinguishes patients with mild to moderate types of OSAHS (distance from the hyoid bone to sella turcica below 120 mm) from patients with a severe type of OSAHS (distance longer than 120 mm). Gungor et al. [21] compared, among others, the position of the hyoid bone in patients diagnosed with OSA and healthy individuals. The position of the hyoid bone was assessed in relation to the mandibular plane. The authors noticed that in patients with OSA, the hyoid bone was significantly lower positioned. Gungor et al. [21] emphasized that the inferiorly positioned hyoid bone is the cause of lower tongue posture, because a larger part of the tongue is moved to the hypopharyngeal area. Consequently, the dimension of airways in OSA patients is significantly decreased comparing to healthy individuals. In our study, we have found that patients with TMD treated with long-term occlusal splint therapy and physiotherapy presented, after the end of the treatment, with a significantly lower positioned hyoid bone and significantly decreased dimension of the lower part of the oropharynx. With reference to the observations by Gungor et al. [21], our results unfortunately indicate that the position of the tongue lowered in our patients.

The possible cause of lowering the position of the hyoid bone and lowering the position of the tongue in patients with TMD treated with long-term occlusal splint therapy and physiotherapy may be the hyperactivity of the superior and middle pharyngeal constrictor muscles [22,23]. We have described in detail the role of increased activity of the superior pharyngeal constrictor muscle in the development of craniovertebral and craniomandibular changes in patients with TMD after long-term occlusal splint therapy and physiotherapy in our previous study [8]. The superior pharyngeal constrictor muscle is attached among others to the mandible and to the tongue [22], whereas the middle pharyngeal constrictor muscle is attached to the hyoid bone [23]. The increased tension of the superior pharyngeal constrictor muscle causes, among other things, the backward and downward rotation of the mandible. Moreover, the glossopharyngeal part of the superior pharyngeal constrictor muscle may lower the position of the tongue [8,22]. Increased tension of the middle pharyngeal constrictor muscle may lower the position of the hyoid bone [23].

Lowering the position of the tongue and a significant decrease in the dimension of the lower part of the oropharynx are the severe side effects of the long-term occlusal splint therapy in patients diagnosed with TMD. This raises the question of whether the long-term occlusal splint therapy is indeed the noninvasive, conservative method of treatment for TMD. Having borne in mind the results of this, as well as our former study [8], the answer to this question is negative.

Our observations clearly indicate that occlusal splint therapy is absolutely contraindicated not only in patients diagnosed with OSA, but also in patients with OSA risk factors. Veasey et al. [24] listed several OSA risk factors in adults, namely: obesity (body mass index greater than 30); male sex; hypothyroidism or acromegaly; increased tonsillar and adenoid tissue; and craniofacial abnormalities, including retrognathia. Veasey et al. [24] also mentioned a few signs and symptoms that may indicate the suspicion of OSA. These are: increased neck circumference (men: larger than 43.2 cm, women: larger than 38.1 cm); crowded oropharynx; snoring, choking and gasping during sleep time; nocturia; sleepiness during the day; headaches in the morning; dry mouth after waking up; and poor quality of sleep no matter how long it lasted. Gulotta et al. [25] discussed the risk factors for OSA in children. The authors mentioned: obesity and overweight, hypertrophy of adenoid and/or tonsils, allergic rhinitis, craniofacial abnormalities, genetics, inflammatory factors, and biomarkers.

It is also recommended to use either the modified Mallampati grade or Friedman tongue position in the assessment of OSA [26,27]. Patients diagnosed with class 3 or even more severe class 4 have been found to present an increased risk of OSA [26,28]. The results of these very easy tests must not be interpreted alone, but only with consideration of anamnesis and clinical examination. In case of suspicion of OSA, either polysomnography or home sleep apnea testing should be performed for the final diagnosis [29].

Patients with OSA or those who have OSA risk factors and at the same time are diagnosed with TMD must not be treated with long-term occlusal splint therapy, because of the above-described side effects. Those patients should be qualified for minimally invasive surgical procedures (arthrocentesis performed alone or in combination with intraarticular injections) [30,31] and/or physiotherapy, depending on the source of pain: TMJs (arthralgia) or adjacent muscles (myalgia).

There are some limitations to the study. Firstly, there is a limited number of participants included in the study. Secondly, patients were included into the groups on the basis of the clinical examinations. There had not been any laboratory tests performed to exclude autoimmune diseases or myopathies. Thirdly, the position of the hyoid bone has been assessed in the two-dimensional cephalometric radiographs. Future studies should include an analysis of 3D images.

## 5. Conclusions

Long-term occlusal splint therapy combined with physiotherapy in patients diagnosed with TMD led to a significant lowering of the hyoid bone position, as well as to a significant decrease in the dimension of the lower part of the oropharynx.

## Figures and Tables

**Table 1:** Reference points, lines, and angles used in cephalometric analysis to assess the position of the hyoid bone and the width of nasopharynx and oropharynx after occlusal splint therapy combined with physiotherapy in patients diagnosed with temporomandibular joint disorders [14,15].

Measurement	Point/Line/Angle	Description
General points and lines	Point ANS	*Anterior nasal spine*–the most prominent point of the anterior nasal spine
	Point PNS	*Posterior nasal spine*–the most posterior point of hard palate, crossed by the pterygopalatine fossa
	Point Pg	*Pogonion*–the most prominent point localized in the mental tuberosity
	Point N	*Nasion*–the most anterior point localized in the frontonasal suture
	Point H	*Hyoidale*–the most superior anterior point of the body of the hyoid bone
	Point C3	The most inferior anterior angle of the body of the third cervical vertebra
	RGN	*Retrognathion*–the most posterior inferior aspect of the mandibular symphysis
	MGP	*McGregor’s Plane*–line which links posterior nasal spine with the basiocciput
Hyoid triangle	H-C3 distance	The distance between the points: H and C3
	H-RGN distance	The distance between the points: H and RGN
	C3-RGN distance	The distance between the points: C3 and RGN
	H-H’ distance	The height of the hyoid triangle, measured as a distance between point H and the perpendicular projection of point H onto C3-RGN line
Hyoid bone topography	H-MGP distance	The distance measured between point H and the perpendicular projection of point H onto MGP
	H-N distance	The distance between the points: hyoidale and nasion
	H-ANS distance	The distance between the points: hyoidale and anterior nasal spine
	H-Pg distance	The distance between the points: hyoidale and pogonion
Airways dimension	Nasopharynx width	The distance measured between posterior nasal spine and the posterior superior point in the nasopharynx
	Oropharynx UP width	The distance measured between the intersection of the functional occlusal plane with the posterior border of the mandibular ramus and the posterior wall of the oropharynx
	Oropharynx LO width	The distance measured between the mandibular angle with the tongue and the posterior wall of the oropharynx

LO—lower, UP—upper.

**Table 2:** The comparison of the examined parameters regarding the hyoid bone position and the width of nasopharynx and oropharynx during the initial examination between study group and control group.

Comparable Characteristic		Study Group av. ± SD (95% CI)	Control Group av. ± SD (95% CI)	*p*–Value
Hyoid triangle	H-C3 distance (mm)	31.8 ± 4.4 (29.9 to 32.6)	33.0 ± 4.8 (30.3 to 35.6)	0.0784 ^b^
	H-RGN distance (mm)	36.3 ± 5.2 (34.4 to 37.8)	39.3 ± 5.7 (36.2 to 42.5)	0.0686 ^a^
	**C3-RGN distance (mm)**	**64.1 ± 8.6 (61.1 to 67.7)**	**70.6 ± 8.3 (66.0 to 75.1)**	**0.0104 ^b^**
	H-H’ distance (mm)	5.5 ± 5.4 (4.2 to 7.1)	5.8 ± 6.4 (2.2 to 9.3)	0.8358 ^b^
Hyoid bone topography	H-MGP distance (mm)	58.0 ± 7.3 (55.4 to 60.9)	57.5 ± 6.6 (53.8 to 61.2)	0.5935 ^b^
	H-N distance (mm)	121.4 ± 8.9 (117.5 to 124.7)	121.2 ± 8.3 (116.7 to 125.8)	0.9145 ^a^
	H-ANS distance (mm)	82.6 ± 8.1 (80.1 to 85.5)	83.2 ± 5.8 (80.0 to 86.4)	0.8343 ^a^
	**H-Pg distance (mm)**	**47.8 ± 6.1 (46.0 to 50.3)**	**51.9 ± 6.6 (48.3 to 55.6)**	**0.0256 ^a^**
Airways dimension	**Nasopharynx width (mm)**	**20.3 ± 3.4 (19.4 to 21.8)**	**23.2 ± 3.2 (21.4 to 25.0)**	**0.0241 ^a^**
	Oropharynx UP width (mm)	10.0 ± 2.3 (9.1 to 11.0)	9.6 ± 2.5 (8.2 to 10.9)	0.4965 ^a^
	Oropharynx LO width (mm)	9.3 ± 2.9 (8.4 to 10.0)	10.3 ± 2.1 (9.1 to 11.4)	0.1868 ^b^

^a^ *t*-Student test, ^b^ U Mann–Whitney test, ANS—anterior nasal spine, C3—third cervical vertebrae, H—hyoidale, H’—the perpendicular projection of point H onto C3-RGN line, MGP—McGregor’s Plane, N—nasion, Pg—pogonion, RGN—retrognathion, UP—upper, LO—lower.

**Table 3:** Assessment of the changes in the hyoid bone position and the width of nasopharynx and oropharynx that occurred between the first and the second orthodontic examination in the control group.

Comparable Characteristic		First Examination av. ± SD (95% CI)	Second Examination (1–2 Years after the Initial One) av. ± SD (95% CI)	*p*–Value
Hyoid triangle	H-C3 distance (mm)	33.0 ± 4.8 (30.3 to 35.6)	33.0 ± 4.8 (30.4 to 35.7)	0.5752 ^a^
	H-RGN distance (mm)	39.3 ± 5.7 (36.2 to 42.5)	39.5 ± 5.9 (36.2 to 42.8)	0.3176 ^a^
	C3-RGN distance (mm)	70.6 ± 8.3 (66.0 to 75.1)	71.0 ± 8.4 (66.4 to 75.6)	0.1820 ^b^
	H-H’ distance (mm)	5.8 ± 6.4 (2.2 to 9.3)	5.7 ± 6.3 (2.2 to 9.2)	0.3464 ^b^
Hyoid bone topography	H-MGP distance (mm)	57.5 ± 6.6 (53.8 to 61.2)	57.5 ± 6.7 (53.8 to 61.2)	0.7557 ^b^
	H-N distance (mm)	121.2 ± 8.3 (116.7 to 125.8)	121.3 ± 8.3 (116.7 to 125.9)	0.5225 ^a^
	H-ANS distance (mm)	83.2 ± 5.8 (80.0 to 86.4)	83.3 ± 5.9 (80.1 to 86.6)	0.4616 ^a^
	H-Pg distance (mm)	51.9 ± 6.6 (48.3 to 55.6)	52.1 ± 6.5 (48.5 to 55.7)	0.1813 ^a^
Airways dimension	Nasopharynx width (mm)	23.2 ± 3.2 (21.4 to 25.0)	23.3 ± 3.2 (21.5 to 25.1)	0.1551 ^a^
	Oropharynx UP width (mm)	9.6 ± 2.5 (8.2 to 10.9)	9.5 ± 2.5 (8.1 to 10.9)	0.0733 ^a^
	Oropharynx LO width (mm)	10.3 ± 2.1 (9.1 to 11.4)	10.3 ± 2.1 (9.1 to 11.4)	0.8447 ^a^

^a^ *t*-Student test, ^b^ Wilcoxon test, ANS—anterior nasal spine, C3—third cervical vertebrae, H—hyoidale, H’—the perpendicular projection of point H onto C3-RGN line, MGP—McGregor’s Plane, N—nasion, Pg—pogonion, RGN—retrognathion, UP—upper, LO—lower.

**Table 4:** Assessment of changes in the hyoid bone position and the width of nasopharynx and oropharynx after occlusal splint therapy combined with physiotherapy in patients diagnosed with temporomandibular joint disorders.

Comparable Characteristic		Before Treatment av. ± SD (95% CI)	After Treatment av. ± SD (95% CI)	*p*–Value
Hyoid triangle	H-C3 distance (mm)	31.8 ± 4.4 (29.9 to 32.6)	31.6 ± 4.8 (29.8 to 33.1)	0.5136 ^b^
	H-RGN distance (mm)	36.3 ± 5.2 (34.4 to 37.8)	36.8 ± 5.4 (34.9 to 38.7)	0.2468 ^a^
	C3-RGN distance (mm)	64.1 ± 8.6 (61.1 to 67.7)	64.6 ± 6.6 (61.7 to 67.0)	0.8453 ^b^
	**H-H’ distance (mm)**	**5.5 ± 5.4 (4.2 to 7.1)**	**7.8 ± 6.4 (5.8 to 9.9)**	**<0.0001 ^b^**
Hyoid bone topography	**H-MGP distance (mm)**	**58.0 ± 7.3 (55.4 to 60.9)**	**61.4 ± 8.2 (58.6 to 64.2)**	**<0.0001 ^a^**
	**H-N distance (mm)**	**121.4 ± 8.9 (117.5 to 124.7)**	**125.5 ± 9.8 (122.5 to 128.3)**	**<0.0001 ^b^**
	**H-ANS distance (mm)**	**82.6 ± 8.1 (80.1 to 85.5)**	**86.4 ± 7.7 (83.9 to 88.6)**	**<0.0001 ^b^**
	H-Pg distance (mm)	47.8 ± 6.1 (46.0 to 50.3)	48.4 ± 5.7 (46.9 to 50.3)	0.2867 ^b^
Airways dimension	Nasopharynx width (mm)	20.3 ± 3.4 (19.4 to 21.8)	20.2 ± 3.1 (19.7 to 20.6)	0.5365 ^a^
	Oropharynx UP width (mm)	10.0 ± 2.3 (9.1 to 11.0)	9.7 ± 2.9 (9.0 to 10.6)	0.2986 ^a^
	**Oropharynx LO width (mm)**	**9.3 ± 2.9 (8.4 to 10.0)**	**8.3 ± 2.6 (7.6 to 9.0)**	**0.0104 ^a^**

^a^ *t*-Student test, ^b^ Wilcoxon test, ANS—anterior nasal spine, C3—third cervical vertebrae, H—hyoidale, H’—the perpendicular projection of point H onto C3-RGN line, MGP—McGregor’s Plane, N—nasion, Pg—pogonion, RGN—retrognathion, UP—upper, LO—lower.

## Data Availability

The data underlying this article are available in the article.
